# Chimeric Flaviviral RNA−siRNA Molecules Resist Degradation by The Exoribonuclease Xrn1 and Trigger Gene Silencing in Mammalian Cells

**DOI:** 10.1002/cbic.202100434

**Published:** 2021-09-12

**Authors:** Cressida Harvey, Sven Klassa, Esteban Finol, Jonathan Hall, Alyssa C. Hill

**Affiliations:** ^1^ Department of Biology ETH Zürich Wolfgang-Pauli-Strasse 27 8093 Zürich Switzerland; ^2^ Department of Chemistry and Applied Biosciences Institute of Pharmaceutical Sciences ETH Zürich Vladimir-Prelog-Weg 1-5/10 8093 Zürich Switzerland

**Keywords:** exonuclease-resistant RNA, RNA nanotechnology, self-assembly, small interfering RNA

## Abstract

RNA is an emerging platform for drug delivery, but the susceptibility of RNA to nuclease degradation remains a major barrier to its implementation *in vivo*. Here, we engineered flaviviral Xrn1‐resistant RNA (xrRNA) motifs to host small interfering RNA (siRNA) duplexes. The xrRNA‐siRNA molecules self‐assemble *in vitro*, resist degradation by the conserved eukaryotic 5’ to 3’ exoribonuclease Xrn1, and trigger gene silencing in 293T cells. The resistance of the molecules to Xrn1 does not translate to stability in blood serum. Nevertheless, our results demonstrate that flavivirus‐derived xrRNA motifs can confer Xrn1 resistance on a model therapeutic payload and set the stage for further investigations into using the motifs as building blocks in RNA nanotechnology.

## Graphical abstract

**Chimeric molecules** comprising an Xrn1‐resistant RNA motif (xrRNA) and a small interfering RNA (siRNA) duplex were engineered. The molecules self‐assemble *in vitro*, resist degradation by the conserved eukaryotic 5’ to 3’ exoribonuclease Xrn1, and trigger gene silencing in mammalian cells. This work sets the stage for further investigations into using xrRNA motifs as building blocks in RNA‐based nanotechnology.

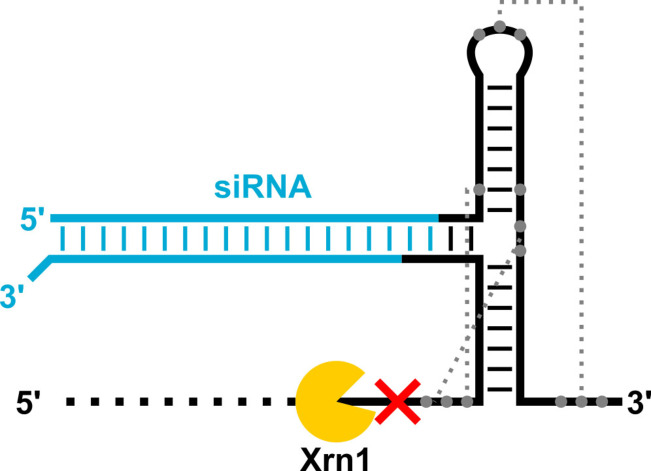

## Introduction

Ribonucleic acid (RNA) is an emerging platform for drug delivery.^[1]^ RNA is biocompatible and exhibits chemical, structural, and functional modularity.^[2]^ Therefore, motifs derived from biological or synthetic RNA molecules can be combined into single, multifunctional RNA structures.^[3]^ Branched RNA molecules,^[4]^ RNA nano‐cubes,^[5]^ and RNA nano‐rings[^5b^, ^6^] have all shown promise as vehicles for the delivery of small interfering RNA (siRNA) duplexes. Their advantages include one‐pot self‐assembly,[^4^, ^5^, ^6^] tissue‐specific delivery,[^4a^, ^4b^, ^4c^, ^4d^, ^4e^, ^4g^] enhanced cellular uptake,[^4g^, ^4h^, ^4i^, ^4j^, ^5b^, ^6b^] alternative processing by the RNA interference (RNAi) machinery,[^4f^, ^4h^, ^4j^] more potent or prolonged RNAi activity,[^4i^, ^4j^, ^4k^, ^5b^] and synergistic or combinatorial RNAi.[^4f^, ^4g^, ^4h^, ^4i^, ^4j^, ^4k^, ^5a^, ^6b^] However, the susceptibility of RNA to nuclease degradation is still a major barrier to its development for applications *in vivo*.

Chemical modification at the base, 2’ position of ribose, or internucleotide linkage is a common nuclease protection strategy for RNA^[7]^ and is widely employed in RNA‐based nanotechnology.^[3]^ For example, RNA molecules that utilize the three‐way junction (3WJ) motif derived from the phi29 bacteriophage prohead RNA (pRNA) are routinely modified with 2’‐deoxyfluoro (2’‐F) substitutions for stability in the biological milieu.[^4a^, ^4b^, ^4c^, ^4d^, ^4e^] However, the incorporation of chemical modifications into therapeutic oligonucleotides can complicate synthesis, reduce potency, and induce cytotoxicity.^[7]^ For example, nucleoside metabolites of 2’‐F oligonucleotides are incorporated into host DNA and RNA and can cause the degradation of paraspeckle proteins.^[8]^ Additionally, the incorporation of 2’‐F modifications into nucleic acid structures sharing the same connectivity, shape, size, charge, and sequences induces the production of proinflammatory cytokines and interferons.^[9]^ Therefore, an attractive alternative would be to identify an RNA motif with intrinsic nuclease resistance and employ that motif as a ‘building block’ in RNA nanotechnology.

Flaviviruses (*e. g*., Dengue virus, West Nile virus, Yellow Fever virus, Zika virus) are small, enveloped viruses with single‐stranded, positive‐sense RNA genomes. They are vectored primarily by arthropods (*e. g*., ticks, mosquitoes) and can cause severe illnesses in humans.^[10]^ A hallmark of flaviviral infection is the accumulation of noncoding subgenomic flaviviral RNA (sfRNA) in infected cells.^[11]^ sfRNA is the product of incomplete degradation of the viral genome by the processive 5’ to 3’ exoribonuclease Xrn1, which is a highly conserved eukaryotic enzyme responsible for most messenger RNA (mRNA) decay in the cytoplasm.^[12]^ Xrn1 partially degrades the viral genome but stalls in the 3’ untranslated region (UTR) when it reaches a complex structure termed Xrn1‐resistant RNA (xrRNA).^[13]^ The xrRNA consists of a 3WJ and pseudoknots, which together adopt a three‐dimensional (3D) ring‐like fold that physically blocks the progression of Xrn1 in the 5’ to 3’ direction.^[14]^ Notably, the xrRNA motif also blocks other, diverse exoribonucleases, including bacterial RNase J1 and yeast Dxo1.^[15]^

Here, we sought to harness the intrinsic nuclease resistance of xrRNA motifs in RNA molecules bearing siRNA duplexes. We designed bipartite xrRNA‐siRNA molecules that self‐assemble *in vitro* from chemically synthesized component strands. The chimeric molecules resist degradation by Xrn1 and trigger gene silencing in 293T cells. However, our data also reveal that the resistance of the molecules to Xrn1 does not translate to stability in blood serum, which may limit the possible applications of xrRNAs *in vivo*. Nevertheless, this work demonstrates that xrRNA motifs can protect a model therapeutic payload from Xrn1‐mediated decay and lays the groundwork for continued investigations into using the motifs as building blocks in RNA nanotechnology.

## Results and Discussion

**Figure 1:**
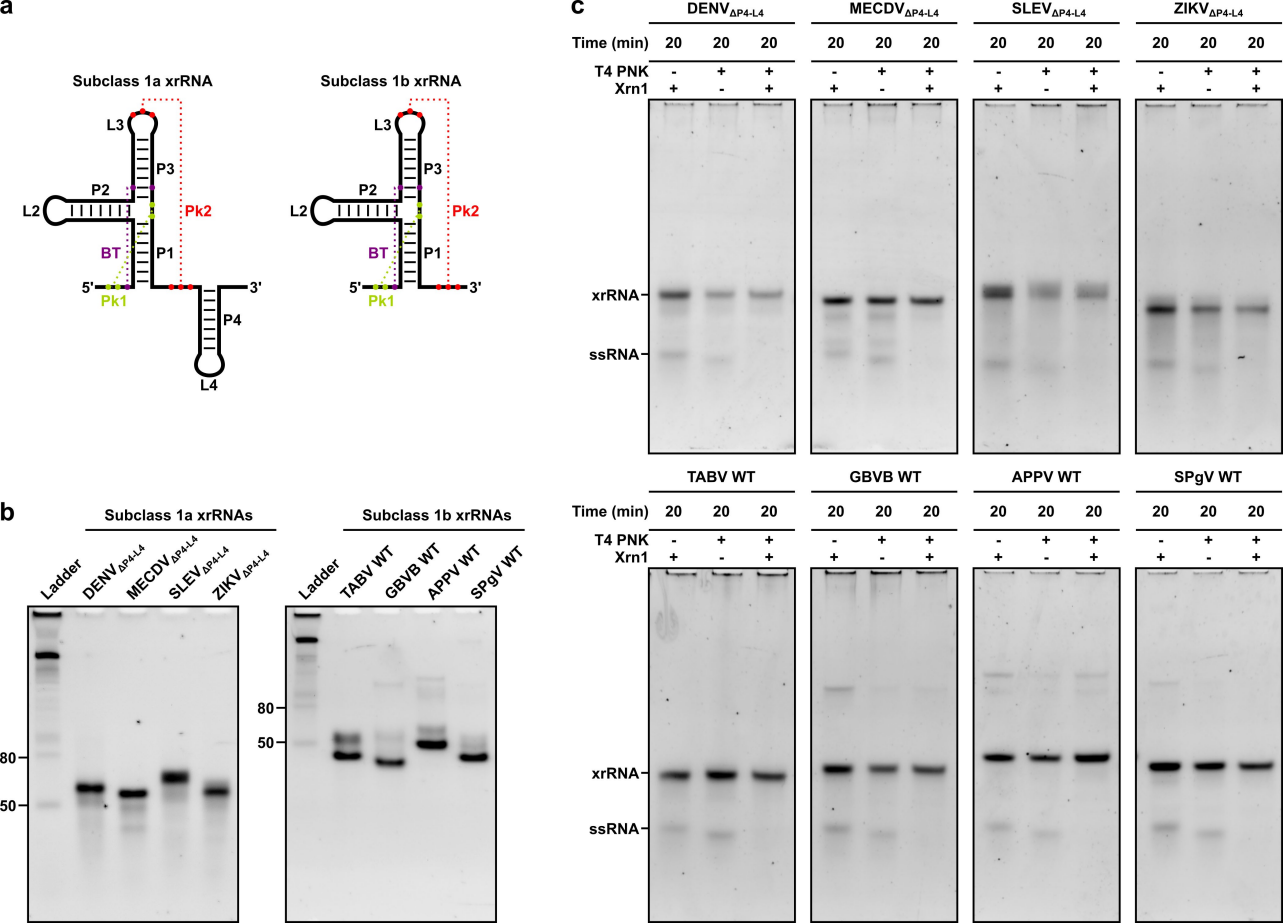
(a) Subclass 1a (left) and 1b (right) xrRNAs. Stems P1 through P4, loops L2 through L4, pseudoknots Pk1 and Pk2, and a base triple (BT) are labelled. Base‐pairing interactions are indicated with dashed lines. (b) Non‐denaturing polyacrylamide gel mobilities of xrRNA constructs. Reference bands in the ladder are single‐stranded RNA. (c) Non‐denaturing polyacrylamide gel mobilities of xrRNA constructs and a single‐stranded RNA (ssRNA) following incubation with Xrn1 at 37 °C for 20 min. Constructs with stem‐loop deletions are denoted by ΔP4−L4. WT, wild type; DENV, Dengue virus; MECDV, Mercadeo virus; SLEV, Saint Louis encephalitis virus; ZIKV, Zika virus; TABV, Tamana bat virus; GBVB, GB virus B; APPV, atypical porcine pestivirus; SPgV, simian pegivirus.

We selected the Dengue virus (DENV), Mercadeo virus (MECDV), Saint Louis encephalitis virus (SLEV), and Zika virus (ZIKV) subclass 1a xrRNA motifs and the Tamana bat virus (TABV), GB virus B (GBVB), atypical porcine pestivirus (APPV), and simian pegivirus (SPgV) subclass 1b xrRNA motifs for initial studies on the basis of 1) short overall length in terms of nucleotides (nt), to maximize yield during solid‐phase synthesis, and 2) number and C : G content of the base‐pairing interactions in the pseudoknot Pk2, to maximize stability. In order to generate minimal subclass 1a xrRNA constructs, we removed the stem‐loop P4−L4, which is not essential for Xrn1 resistance^[16]^ and is naturally absent from many subclass 1b xrRNA motifs (Figure 1a).^[17]^ Oligoribonucleotides were produced by solid‐phase synthesis (Supporting Information Table S1). Yield and purity were verified by liquid chromatography‐mass spectrometry (LC‐MS) (Supporting Information Table S2). To assess their native gel mobilities, we folded the xrRNA constructs at 200 nM in 100 mM NaCl, 10 mM MgCl_2_, 50 mM Tris, 1 mM DTT, pH 7.9 and subjected them to non‐denaturing polyacrylamide gel electrophoresis (PAGE). The subclass 1a xrRNAs migrated as a dominant species in the 50–80 nt range, while the subclass 1b xrRNAs migrated as one or two dominant species around 50 nt (Figure 1b).

To determine whether the designed xrRNA constructs adopt their native 3D fold, we assayed for Xrn1 resistance. Xrn1 requires a 5’‐terminal phosphate for activity.^[18]^ Therefore, we carried out 5’ phosphorylation of the oligoribonucleotides, which bear a 5’‐terminal hydroxyl group following solid‐phase synthesis, using T4 polynucleotide kinase (PNK). As an internal positive control, we spiked in an unrelated, short (21‐nt), single‐stranded RNA (*i. e*., siREN AS; Supporting Information Table S3), which was not expected to interact with the xrRNA sequences by base complementarity. Following 5’ phosphorylation, the oligoribonucleotides were purified on Microspin^TM^ G‐25 columns (Sigma‐Aldrich), folded in 100 mM NaCl, 10 mM MgCl_2_, 50 mM Tris, 1 mM DTT, pH 7.9, and incubated with Xrn1 at 37 °C for 20 min. A non‐phosphorylated 21‐mer and xrRNA construct pair also were incubated with Xrn1 at 37 °C for 20 min. All samples were subjected to non‐denaturing PAGE. Xrn1 resistance was assessed by comparing the band intensities of 5’‐phosphorylated RNAs incubated without (−) and with (+) Xrn1, where a decrease in band intensity was attributed to degradation. A decrease in band intensity between non‐phosphorylated and 5’‐phosphorylated RNAs was attributed to sample loss during the column purification step. The 5’‐phosphorylated 21‐mers were degraded within 20 min, while the 5’‐phosphorylated xrRNAs remained intact over this period (Figure 1c). These results indicate that the designed xrRNA constructs adopt their native, Xrn1‐resistant 3D fold and further confirm that the stem‐loop P4−L4 is not required for Xrn1 resistance.

**Figure 2:**
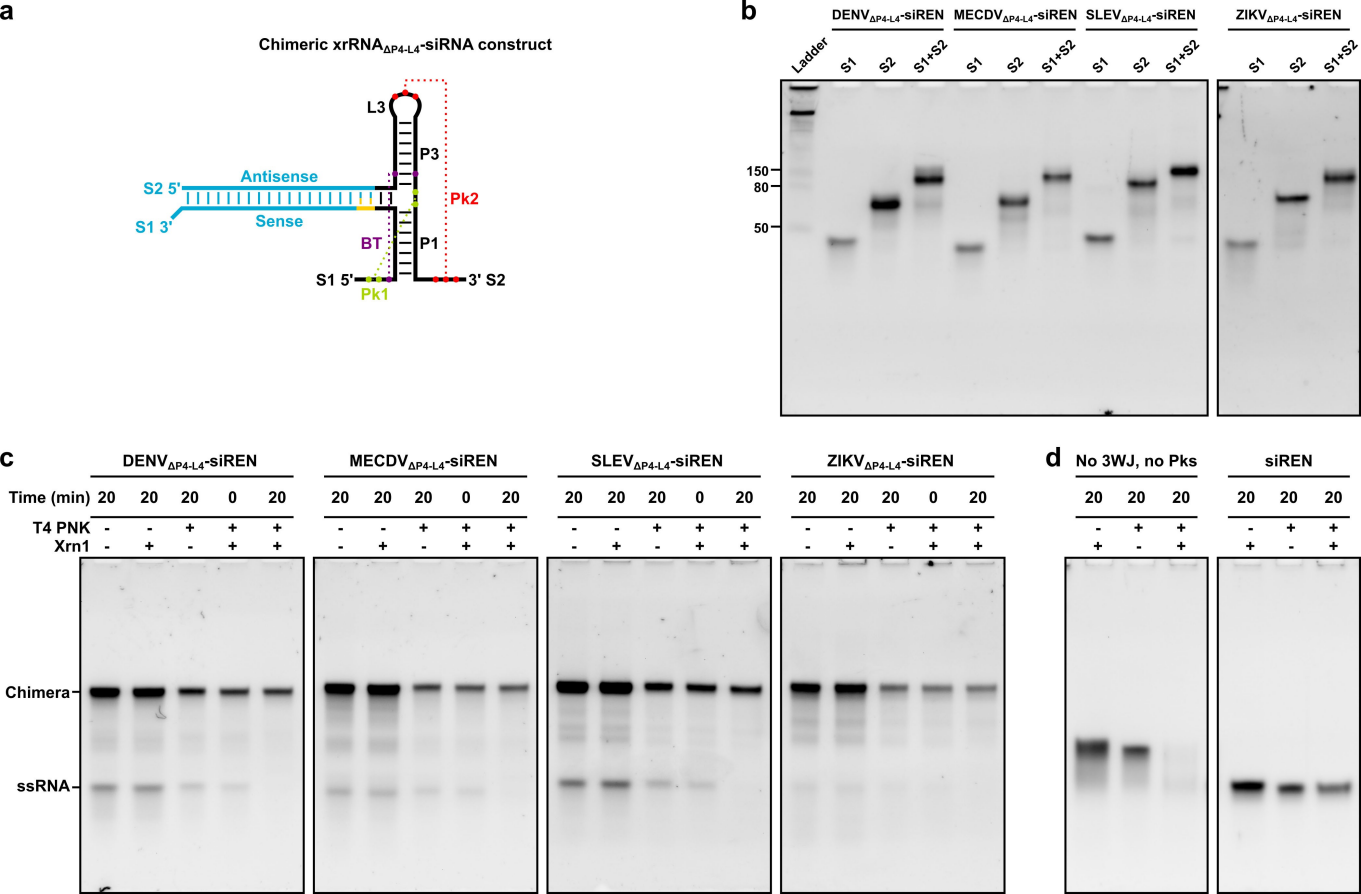
(a) Chimeric xrRNA_ΔP4−L4_−siRNA construct. The stem‐loop P2‐L2 of the subclass 1a xrRNA shown in Figure 1 was exchanged with an siRNA (blue). Antisense and sense sequences, strand 1 (S1) and strand 2 (S2), stems P1 and P3, loop L3, pseudoknots Pk1 and Pk2, and a base triple (BT) are labelled. Base‐pairing interactions are indicated with dashed lines. An adapter is yellow. (b) Non‐denaturing polyacrylamide gel mobilities of xrRNA_ΔP4−L4_−siRNA constructs. Reference bands in the ladder are single‐stranded RNA. (c) Non‐denaturing polyacrylamide gel mobilities of xrRNA_ΔP4−L4_−siRNA constructs with a two‐fold molar excess of S1 to S2 following incubation with Xrn1 at 37 °C for 20 min. The excess S1 is labelled as a single‐stranded RNA (ssRNA). (d) Non‐denaturing polyacrylamide gel mobilities of a control comprising DENV_ΔP4−L4_−siREN S1 and siREN AS, which lacks the xrRNA 3WJ and pseudoknots, and a control siRNA (siREN) following incubation with Xrn1 at 37 °C for 20 min.

Next, we exchanged the stem‐loop P2‐L2 with an siRNA targeting Renilla luciferase (siREN) or a non‐targeting control siRNA (siRND) for use in downstream dual‐luciferase reporter assays. The subclass 1a xrRNAs were selected as a representative subset for engineering. siRNAs are the canonical exogenous triggers of RNAi with 19 base‐pair duplexes and dinucleotide 3’ overhangs.^[19]^ To accommodate one overhang, we added a dinucleotide adapter to our xrRNA‐siRNA designs. The adapter base pairs with the overhang to ensure there are no unpaired nucleotides at the xrRNA:siRNA fusion site. With the inclusion of the siRNA, two separate strands were generated: strand 1 (S1; approximately 30 nt) and strand 2 (S2; approximately 60 nt) (Figure 2a). The sense sequence was embedded in S1, and the antisense sequence was embedded in S2, in line with data showing that this orientation yields molecules with higher gene silencing potency (data not shown). The antisense and sense sequences of siRND each bear six randomized base‐pair positions, as described previously.^[20]^ The single‐stranded randomized RNAs hybridize to form their most thermodynamically stable (*i. e*., complementary) duplexes^[20]^ and therefore were not expected to interfere with the formation of the xrRNA‐siRNA structure. Oligoribonucleotides were produced by solid‐phase synthesis (Supporting Information Table S3). Yield and purity were verified by LC‐MS (Supporting Information Table S4). To assess whether the constructs self‐assemble, we annealed S1 and S2 at 200 nM in 100 mM NaCl, 10 mM MgCl_2_, 50 mM Tris, 1 mM DTT, pH 7.9 and subjected them to non‐denaturing PAGE. For each construct, S1 migrated below 50 nt, and S2 migrated around 80 nt (Figure 2b). Together, S1 and S2 exhibited a reduced mobility relative to either S1 or S2 alone (Figure 2b), which is consistent with the formation of an xrRNA‐siRNA construct. All xrRNA‐siRNA constructs migrated as a dominant species in the 80–150 nt range (Figure 2b, Supporting Information Figure S1). Interestingly, the constructs exhibited similar mobilities under denaturing conditions (Supporting Information Figure S2), consistent with an ability to resist urea denaturation, which has been reported for RNA molecules based on the phi29 bacteriophage pRNA 3WJ.^[4a]^

To determine whether the xrRNA‐siRNA constructs resist degradation by Xrn1, we assayed for Xrn1 resistance as described above. Briefly, the oligoribonucleotides were 5’ phosphorylated, purified on Microspin^TM^ G‐25 columns (Sigma‐Aldrich), annealed in 100 mM NaCl, 10 mM MgCl_2_, 50 mM Tris, 1 mM DTT, pH 7.9, and incubated with Xrn1 at 37 °C for 20 min. As an internal positive control, we used a two‐fold molar excess of S1 to S2. Degradation was analyzed by non‐denaturing PAGE as described above. The 5’‐phosphorylated 30‐mers were degraded within 20 min, while the 5’‐phosphorylated xrRNA‐siRNAs remained intact over this period (Figure 2c). In fact, the xrRNA‐siRNA molecules resisted Xrn1 degradation for up to 20 h, the longest time point tested (Supporting Information Figure S3). To confirm that xrRNA‐siRNAs stop an actively degrading enzyme, we carried out an Xrn1 resistance assay on a DENV_ΔP4−L4_−siREN construct with a 31‐nt, unstructured “leader” sequence at the 5’ end of S1 (Supporting Information Tables S3, S4 and Figure S4). Incubation of this construct with Xrn1 yielded a degradation‐resistant product that persisted for up to 20 h (Supporting Information Figure S4). A control comprising DENV_ΔP4−L4_−siREN S1 and siREN AS, which lacks the xrRNA 3WJ and pseudoknots, was degraded within 20 min (Figure 2d). A fraction of a control siRNA (*i. e*., siREN) incubated with Xrn1 remained intact after 20 min (Figure 2d), suggesting that sequestration of the 5’ end of an RNA plays a role in preventing Xrn1 loading and subsequent degradation.

**Figure 3:**
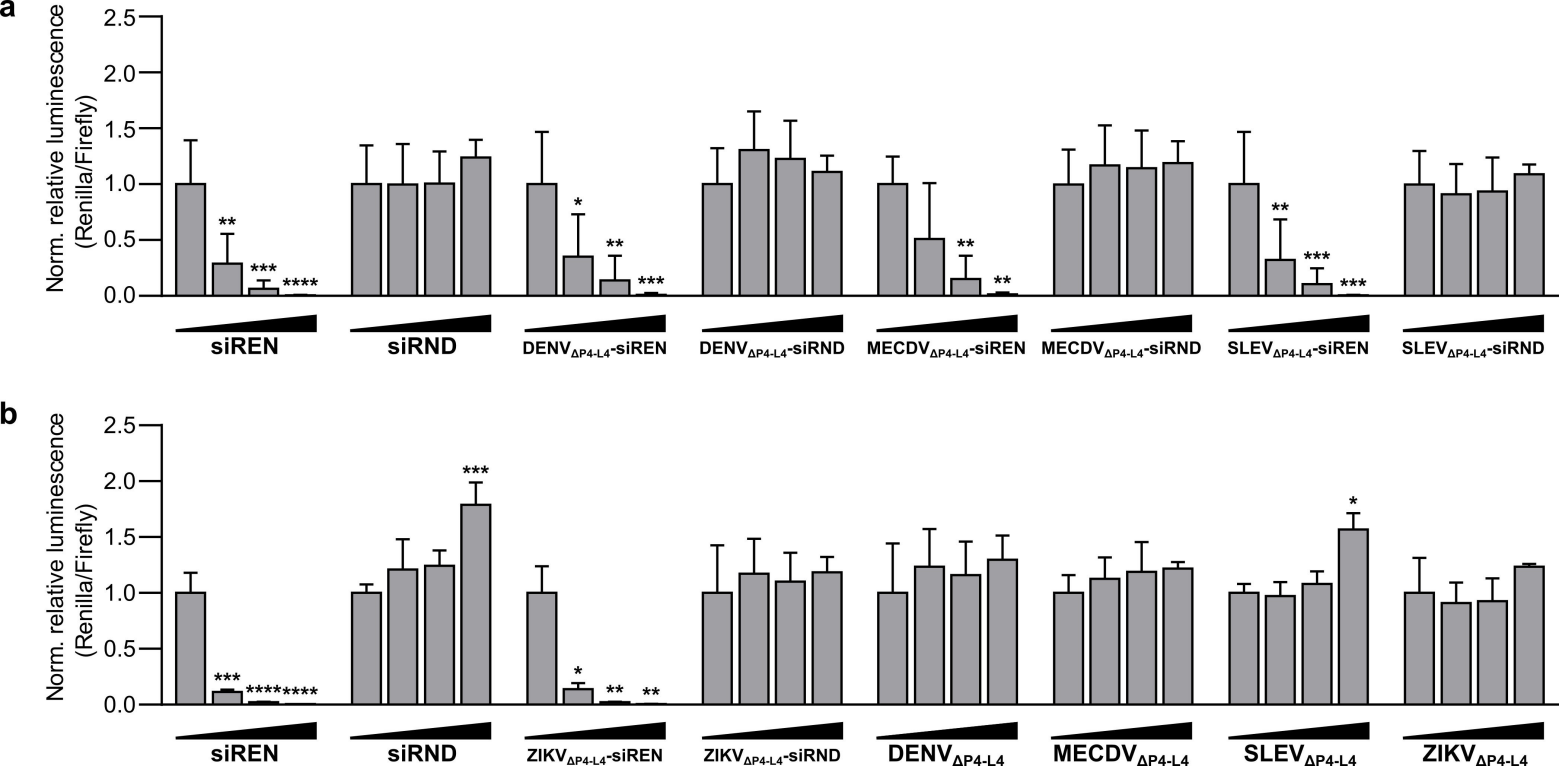
(a) Activity of xrRNA_ΔP4−L4_−siRNA constructs on a Renilla luciferase reporter in 293T cells. (b) Activity of xrRNA_ΔP4−L4_−siRNA constructs and xrRNA_ΔP4−L4_ constructs on the same reporter in 293T cells. siREN and siRND served as positive and negative controls, respectively. All treatments were transfected at 0 nM, 2.5 nM, 10 nM, and 40 nM. A difference in the silencing efficiency of siREN between (a) and (b) was attributed to a difference in transfection efficiency. The antisense and sense sequences of siRND each bear six randomized base‐pair positions, as described previously.^[20]^ Data are mean relative luminescence (Renilla/Firefly) values normalized to the 0 nM treatment±SD (n=3). Statistics are two‐way ANOVA with Dunnett's multiple comparisons test: *P≤0.05, **P≤0.01, ***P≤0.001, ****P≤0.0001.

To assess whether the xrRNA‐siRNA constructs trigger gene silencing, we performed dual‐luciferase reporter assays. Briefly, S1 and S2 were annealed at 20 μM in 100 mM NaCl, 10 mM MgCl_2_, 50 mM Tris, 1 mM DTT, pH 7.9 and transfected into 293T cells over a concentration range. siREN and siRND were transfected into 293T cells over the same concentration range and served as positive and negative controls, respectively. A plasmid expressing both Renilla and Firefly luciferase was introduced into the cells 24 h later. At 48 h post‐plasmid transfection, luminescence counts for both Firefly and Renilla luciferase were measured. Remarkably, all xrRNA‐siREN constructs triggered dose‐dependent silencing of Renilla luciferase, while the xrRNA‐siRND constructs were inactive (Figure 3). The xrRNA constructs alone also were inactive (Figure 3), further confirming that the gene silencing activity observed for the xrRNA‐siREN constructs is specific to the siREN moiety. Notably, all xrRNA‐siREN constructs were as potent as the naked siREN molecule (two‐way ANOVA with Tukey's multiple comparisons test, α=0.05).

**Figure 4:**
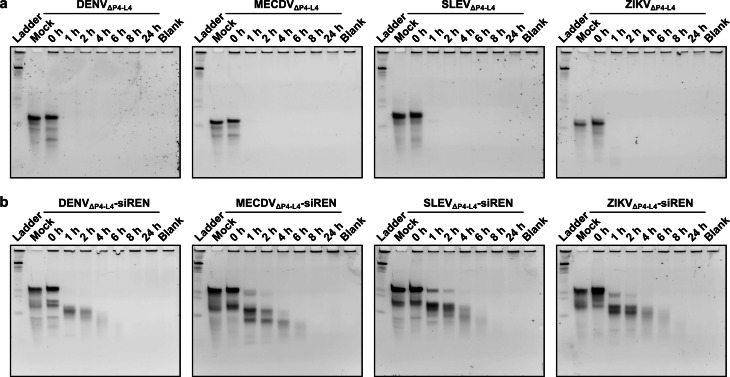
(a) Serum stability assays of xrRNA_ΔP4−L4_ constructs. (b) Serum stability assays of xrRNA_ΔP4−L4_−siRNA constructs. The length of incubation in 50 % mouse serum at 37 °C is indicated above each lane. Mock is RNA incubated in water at 37 °C for 24 h. Blank is 50 % mouse serum incubated at 37 °C for 24 h. Reference bands in the ladder are single‐stranded RNA.

To test whether Xrn1 resistance might translate to stability in blood serum, we performed serum stability assays. Briefly, constructs were folded or annealed in 100 mM NaCl, 10 mM MgCl_2_, 50 mM Tris, 1 mM DTT, pH 7.9 and then mixed 1 : 1 with mouse serum prior to incubation at 37 °C. As controls, a mock sample containing RNA in water and a blank sample containing 50 % mouse serum also were incubated at 37 °C. Degradation was analyzed by non‐denaturing PAGE. Most xrRNA constructs were degraded within 1 h (Figure 4a). Likewise, most xrRNA‐siRNA constructs were degraded within 1 h (Figure 4b). Similar results were obtained in a different batch of mouse serum (Supporting Information Figure S5). In general, the subclass 1b xrRNAs were not more stable in serum than the subclass 1a xrRNAs (Supporting Information Figure S6). However, among all xrRNA constructs, the GBVB construct displayed the highest apparent stability, which may derive from its specific primary and secondary structure. Among the xrRNA‐siRNA constructs, the SLEV_ΔP4−L4_−siREN construct displayed the highest apparent stability. Interestingly, for one construct we achieved a higher apparent stability by re‐installing the stem‐loop P4−L4 (Supporting Information Table S5 and Figure S7) and annealing S1 and S2 in 1 M NaCl, 10 mM cacodylic acid, pH 7.0. Indeed, a fraction of the MECDV−siREN construct prepared in this manner remained intact in serum for up to 8 h (Supporting Information Figure S7). We attempted to further stabilize this construct through mutation of Pk2 to all C : G base pairs (Supporting Information Table S6 and Figure S8). Surprisingly, however, the mutant MECDV−siREN constructs were less stable in serum than the parent construct (Supporting Information Figure S8). Further studies will be required to elucidate the relationship among xrRNA sequence, structure, and stability.

## Conclusion

This study engineered flavivirus‐derived xrRNA motifs to host siRNA duplexes. The molecules self‐assembled *in vitro* from chemically synthesized component strands, and they resisted degradation by the highly conserved eukaryotic 5’ to 3’ exoribonuclease Xrn1. Moreover, the molecules triggered silencing of a Renilla luciferase reporter in 293T cells with a potency comparable to a naked siRNA molecule. We speculate that siRNAs are released from xrRNA‐siRNAs by Dicer, which has been reported for siRNAs fused to the phi29 bacteriophage pRNA 3WJ,^[4d]^ RNA nano‐cubes,^[5]^ and RNA nano‐rings.[^6a^, ^6b^] However, it is possible that xrRNA‐siRNAs do not require Dicer to induce gene silencing, which has been reported for several synthetic RNAi triggers.^[21]^ Nevertheless, our results indicate that the stability of the molecules to Xrn1 does not translate to stability in blood serum. RNase 1 is a member of the RNase A superfamily and accounts for approximately 70 % of the RNase activity in human blood serum.^[22]^ Moreover, inhibition of RNase A enzymes has been shown to prolong siRNA half‐life in both human and mouse blood serum.^[23]^ Therefore, we speculate that stabilizing xrRNAs to RNase A enzymes, and RNase 1 specifically, will be required to advance the motifs as drug delivery vehicles *in vivo*. To this end, xrRNA crystal structures[^13^, ^14^, ^24^] and chemical probing data^[17]^ may facilitate the rational placement of chemical modifications in these motifs.

Although xrRNAs may not be well suited to protect double‐stranded siRNAs from extracellular nucleases, our results provide a foundation for further investigations into using the motifs as building blocks in RNA nanotechnology. In particular, mRNA payloads may benefit from protection against Xrn1, whose major role is the degradation of mRNA following decapping. In *S. cerevisiae*, DENV xrRNAs placed upstream of a LacZ reporter and internal ribosome entry site (IRES) resulted in a decay‐resistant mRNA construct that increased β‐galactosidase activity 30‐fold.^[25]^ A similar approach could boost the half‐life, and possibly efficacy, of therapeutic mRNAs, including mRNA‐based vaccines. Xrn1 is also known to digest other RNA substrates, including long noncoding RNAs (lncRNAs), transfer RNAs (tRNAs), ribosomal RNAs (rRNAs), and small nucleolar RNAs (snoRNAs).^[26]^ Therefore, these and other RNA payloads (*e. g*., aptamers, ribozymes) may also benefit from protection against Xrn1 by xrRNA motifs. Finally, structure‐based mechanisms to resist decay have been identified in other naturally occurring RNAs, including lncRNAs found in Kaposi's sarcoma‐associated herpesvirus and tRNA‐like sequences in plant viruses.^[27]^ Undoubtedly, additional studies will be required to investigate the potential of these unique, degradation‐resistant structures in RNA nanotechnology.

## Experimental Section

**Oligoribonucleotide synthesis and purification**: Oligoribonucleotide synthesis was carried out on the small scale (50 nmol) on a MerMade 12 synthesizer (BioAutomation Corporation) in DMT‐on mode. For each synthesis, we used approximately 5 mg UnySupport controlled‐pore glass (CPG; Glen Re‐search) solid support with a pore size of 500 Å, 1000 Å, or 2000 Å depending on sequence length. We used nucleoside phosphoramidites corresponding to ribonucleotides A, C, G, and U (Thermo Scientific). An equimolar mixture of A, C, G, and U phosphoramidites was used for randomized nucleotide positions, as described previously.^[20]^ RNA was cleaved from the solid support with gaseous methylamine (PanGas) at 70 °C and 1.2 bar for 1.5 h or in a solution of ammonium hydroxide (28 %; Sigma‐Aldrich) and methylamine (40 wt. % in H_2_O; Sigma‐Aldrich) (AMA; 1 : 1) at 65 °C for 45 min in an Eppendorf ThermoMixer C. The RNA was eluted with ethanol/water (1 : 1) and dried in a vacuum concentrator (Christ RVC 2‐25CD) at 35–45 °C until solid. Silyl deprotection was carried out using a solution of freshly prepared dry 1‐N‐methyl‐2‐pyrrolidone (97 %; Acros Organics), triethylamine (99 %; Merck), and triethylamine trihydrofluoride (97 %; Acros Organics) (6 : 3 : 4) at 70 °C for 2 h in an Eppendorf ThermoMixer C. The reaction was quenched with trimethylethoxysilane (98 %; Tokyo Chemical Industry) and RNA was precipitated with diisopropyl ether (99 %; Sigma‐Aldrich). RNA pellets were dissolved in ultrapure water (Synergy® UV, Merck Millipore) and purified by reverse‐phase high performance liquid chromatography (RP‐HPLC; Agilent 1200 Series, Agilent Technologies). For DMT‐on purification, we used the Waters XBridge OST C18 column (10×50 mm, 2.5 μm pore size) heated to 65 °C and a gradient of 20–60 % HPLC grade acetonitrile (99.9 %; Sigma‐Aldrich) in 0.1 M triethylammonium acetate (TEAA) buffer, pH 8.0, in 5 min with a flow rate of 5 mL/min. The main fractions were pooled and dried in a vacuum concentrator as described above. DMT deprotection was carried out with the addition of 20 % (v/v) acetic acid (99.8 %; VWR Chemicals) at 25 °C for 15 min. The acetic acid was evaporated in a vacuum concentrator as described above and RNA pellets were dissolved in ultrapure water for final, DMT‐off purification using a gradient of 10–35 % acetonitrile in TEAA buffer, pH 8.0. The main fractions were pooled, dried in a vacuum concentrator as described above, and dissolved in ultrapure water. The RNA concentration of each sample was calculated according to the Beer‐Lambert law using the absorbance measured at 260 nm on a Nanodrop 2000 (Thermo Scientific) and the extinction coefficient provided by the online tool OligoAnalyzer (Integrated DNA Technologies). LC‐MS analysis was carried out by injecting 0.5 nmol of each oligoribonucleotide in 20 μL ultrapure water into an Agilent 6130 Series Quadrupole LC/MS (Agilent Technologies) with electron spray ionization.

**PAGE analysis**: For all PAGE experiments, we used Mini‐PROTEAN® Spacer Plates, 0.75 mm (Bio‐Rad) and Mini‐PROTEAN® Combs, 10‐well, 0.75 mm (Bio‐Rad) to cast the gels and the Mini‐PROTEAN® Tetra Cell (Bio‐Rad) with the Mini‐PROTEAN® PowerPac^TM^ Basic Power Supply (Bio‐Rad) to run the gels. For non‐denaturing PAGE analysis, 12 % PAGE gels were prepared using a 30 % acrylamide/bis‐acrylamide (29 : 1) solution (Bio‐Rad) in Tris/borate/EDTA (TBE) buffer (Acros Organics). Polymerization was initiated with the addition of freshly prepared 10 % (v/v) ammonium persulfate (APS; 98 %; Sigma‐Aldrich) and NNN'N’‐tetramethylethylenediamine (TEMED; 97 %; Fisher Scientific). Polymerized gels were pre‐run at 4 °C and 100 V in 0.5× TBE buffer for 30 min. Prior to loading, samples were mixed with Gel Loading Dye, Purple, no SDS (New England Biolabs). We used the Short Range ssRNA Ladder (New England Biolabs) as a band size reference. The gels were run at 4 °C and 100 V in 0.5× TBE buffer for 2 h. RNA was stained with SybrGold (Invitrogen) and visualized by UV transillumination on a ChemiDoc XRS+ Imager (Bio‐Rad). For denaturing PAGE analysis, 12 % denaturing gels were prepared according to the SequaGel® UreaGel^TM^ System (National Diagnostics) instructions. Gels were pre‐run at room temperature and 150 V in 0.5× TBE buffer for 20 min. Samples and the ladder were prepared as described above. The gels were run at room temperature and 200 V for 50 min. Staining and visualization of RNA was performed as described above.

**Xrn1 resistance assay**: For xrRNA constructs, 5’ phosphorylation was achieved by preparing 150 ng xrRNA and 150 ng siREN antisense strand in T4 PNK Buffer A (Thermo Fisher) with 1 mM ATP (Thermo Fisher) and 20 U T4 PNK (Thermo Fisher) in 40 μL. The reactions were incubated at 37 °C for 20 min and then heat inactivated at 75 °C for 10 min in an Eppendorf ThermoMixer Comfort. Samples were purified on Microspin^TM^ G‐25 columns (Sigma‐Aldrich), dried in a vacuum concentrator (Christ RVC 2‐25CD) at 45 °C, and resuspended in 14 μL Xrn1 buffer (100 mM NaCl, 10 mM MgCl_2_, 50 mM Tris, 1 mM DTT, pH 7.9). A non‐phosphorylated sample containing 100 ng xrRNA and 100 ng siREN antisense strand also was prepared in 14 μL Xrn1 buffer. All samples were folded by heating at 90 °C for 1 min, cooling to 20 °C and holding for 5 min, and cooling to 4 °C in a thermal cycler (C1000 Touch^TM^ Thermal Cycler; Bio‐Rad). The 5’ phosphorylated RNA was split evenly between two tubes. Into one tube, we added 1 μL Xrn1 (1 U/μL; New England Biolabs) and brought the reaction to 15 μL with Xrn1 buffer; into the other, we added no enzyme and brought the reaction to 15 μL with Xrn1 buffer. To the 200 ng non‐phosphorylated RNA, we added 0.66 μL Xrn1 (1 U/μL; New England Biolabs) and brought the reaction to 20 μL with Xrn1 buffer. All three reactions had a final RNA concentration of 10 ng/uL and final Xrn1 concentration of 0.33 U/uL. Following the addition of Xrn1, the samples were incubated at 37 °C and 300 rpm for 20 min and then heat inactivated at 70 °C for 10 min in an Eppendorf ThermoMixer Comfort. Samples were stored overnight at −80 °C. The following day, 5 μL of each sample was run on a 12 % non‐denaturing PAGE gel as described above. For xrRNA‐siRNA constructs, 5’ phosphorylation was achieved by preparing 13 pmol S1 and 6.5 pmol S2 in T4 PNK Buffer A (Thermo Fisher) with 1 mM ATP (Thermo Fisher) and 10 U T4 PNK (Thermo Fisher) in 20 μL. The reactions were incubated at 37 °C for 20 min and then heat inactivated at 75 °C for 10 min in an Eppendorf ThermoMixer Comfort, purified on Microspin^TM^ G‐25 columns (Sigma‐Aldrich), dried in a vacuum concentrator as described above, and then resuspended in 7 μL Xrn1 buffer (100 mM NaCl, 10 mM MgCl_2_, 50 mM Tris, 1 mM DTT, pH 7.9). A non‐phosphorylated sample containing 8.6 pmol S1 and 4.3 pmol S2 also was prepared in 7 μL Xrn1 buffer. All samples were annealed by heating at 90 °C for 1 min, cooling to 20 °C and holding for 5 min, and cooling to 4 °C in a thermal cycler (C1000 Touch^TM^ Thermal Cycler; Bio‐Rad). To 5’ phosphorylated RNAs, we added 1 μL Xrn1 (1 U/μL; New England Biolabs) and brought the reaction to 32 μL with Xrn1 buffer. To the non‐phosphorylated sample, we added 0.33 μL Xrn1 (1 U/μL; New England Biolabs) and brought the reaction to 10 μL with Xrn1 buffer. Following the addition of Xrn1, all samples were incubated at 37 °C and 300 rpm in an Eppendorf ThermoMixer Comfort. At 0 min, 5 min, 30 min, 1 h, 3.5 h, and 20 h, 5 μL aliquots were heat inactivated at 70 °C for 10 min and then stored overnight at −80 °C. The following day, 5 μL of each sample was run on a 12 % non‐denaturing PAGE gel as described above.

**Dual‐luciferase reporter assay**: 293T cells were grown in Dulbecco's Modified Eagle's Medium (DMEM; Thermo Fisher) supplemented with 10 % fetal bovine serum (FBS; Sigma‐Aldrich) at 37 °C with 5 % CO_2_ in a humidified incubator. The cells were seeded at a density of 10,000 live cells/well in DMEM+10 % FBS in opaque, 96‐well plates. Approximately 8 h post‐seeding, constructs and controls folded or annealed at 20 μM in 100 mM NaCl, 10 mM MgCl_2_, 50 mM Tris, 1 mM DTT, pH 7.9 were transfected into the cells in technical triplicate at final concentrations of 0 nM, 2.5 nM, 10 nM, or 40 nM per well using Lipofectamine 2000 transfection reagent (Thermo Fisher Scientific) according to the manufacturer's instructions. Approximately 24 h later, a reporter plasmid expressing both Firefly and Renilla luciferase was transfected into the cells at 20 ng/well using jetPEI^TM^ transfection reagent (Polyplus) according to the manufacturer's instructions. Luminescence counts were measured 48 h post‐plasmid transfection on a Mithras LB 940 luminometer (Berthold Technologies) using the Dual‐Glo® Luciferase Assay System (Promega) according to the manufacturer's instructions.

**Serum stability assay**: RNA samples were prepared at 1.6 μM in 100 mM NaCl, 10 mM MgCl_2_, 50 mM Tris, 1 mM DTT, pH 7.9 or 1 M NaCl, 10 mM cacodylic acid, pH 7.0 and folded or annealed by heating at 90 °C for 2 min, cooling to 20 °C and holding for 5 min, and cooling to 4 °C in a thermal cycler (C1000 Touch^TM^ Thermal Cycler; Bio‐Rad). Samples diluted to 0.62 μM were mixed 1 : 1 with mouse serum (Sigma‐Aldrich) or water (mock). A blank sample containing serum/water (1 : 1) also was prepared. All samples were incubated at 37 °C and 300 rpm in an Eppendorf ThermoMixer Comfort. Aliquots taken at 0 h, 1 h, 2 h, 4 h, 6 h, 8 h, and 24 h and mock and blank samples taken at 24 h were mixed with Tissue and Cell Lysis Solution (Lucigen) and 4 μL Proteinase K (20 mg/mL; Sigma‐Aldrich), incubated at 65 °C for 30 min, and stored overnight at −80 °C. After thawing on ice, samples were heat denatured at 95 °C for 5 min, and SDS was precipitated with 3 M KCl at room temperature and 1200 rpm for 10 min in an Eppendorf ThermoMixer Comfort. SDS was pelleted by centrifugation at 4 °C and 12,000×*g* for 10 min (Heraeus Fresco 17 Centrifuge, Thermo Scientific). Supernatants were transferred into fresh tubes and 10 μL of each sample was run on a 12 % non‐denaturing PAGE gel as described above.

## Conflict of interest

The authors declare no conflict of interest.

## Supporting information

As a service to our authors and readers, this journal provides supporting information supplied by the authors. Such materials are peer reviewed and may be re‐organized for online delivery, but are not copy‐edited or typeset. Technical support issues arising from supporting information (other than missing files) should be addressed to the authors.

Supporting Information
