# Supplementary material for: Chimeric Flaviviral RNA−siRNA Molecules Resist Degradation by The Exoribonuclease Xrn1 and Trigger Gene Silencing in Mammalian Cells
Source: Chembiochem. 2021 Sep 12;22(21):3099–106. doi: 10.1002/cbic.202100434 (PMC8596575; doi:10.1002/cbic.202100434)
Supplement: Supplementary file 1 — Supporting Information [file CBIC-22-3099-s001.pdf]

ChemBioChem

Supporting Information

Chimeric Flaviviral RNA–siRNA Molecules Resist Degradation by The Exoribonuclease Xrn1 and Trigger Gene Silencing in Mammalian Cells

Cressida Harvey, Sven Klassa, Esteban Finol, Jonathan Hall, and Alyssa C. Hill*

Table S1. Sequences of xrRNA constructs. Constructs with stem-loop deletions are denoted by ΔP4-L4. WT, wild type; DENV, Dengue virus; MECDV, Mercadeo virus; SLEV, Saint Louis encephalitis virus; ZIKV, Zika virus; TABV, Tamana bat virus; GBVB, GB virus B; APPV, atypical porcine pestivirus; SPgV, simian pegivirus.

Entry	Sequence (5' to 3')	Accession number	Range
$DENV_{ΔP4-L4}$	AGUCAGGCCGGAUUAAGCCAUAGUACGGUAAGAGCUAUGCUGCCUGUGAGCCC	MT447147	10324-10376
$MECDV_{ΔP4-L4}$	AGUCAGACCGAAAAGCCACUCAAUGGUUGUAAAGAGUGCUGUCUGUGACAAC	NC_027819	10594-10642
$SLEV_{ΔP4-L4}$	AGUCAGGCCAAUCAGUUUUGCCACCGGAUGUCAGGUAAACGGUGCUGUCUGUAACCUGGC	NC_007580.2	10430-10489
$ZIKV_{ΔP4-L4}$	AGUCAGGCCGAUAACGCCAUGGCACGGAAAAAGCCAUGCUGCCUGUGAGCC	MF510857.1	10476-10526
TABV WT	GGCAAGGUACGGAUUAGCCGUAGGGGCUUGAGAACCCCCCCUCCCCACUC	AF346759	10226-10275
GBVB WT	GGCAACAGGGGAGACCCCGGGCUUAACGACCCCGCCGAUGUGAGUU	NC_001655.1	9099-9144
APPV WT	GGGCAAGUGGCCGUAUAGGCUGGGGCGAUCGCCGUACCCCCCCUUUACCAGGCG	NC_038964.1	11108-11161
SPgV WT	GGCAGCAGCAGACGCAAGUCUGGGGGAAACGAUCGCUCCUCCCUCUGCAGAUC	U94421.1	9446-9498

Table S2. Masses calculated and found for xrRNA constructs.

Entry	Mass calculated (g/mol)	Mass found (g/mol)
$DENV_{ΔP4-L4}$	17076.3	17076.45
$MECDV_{ΔP4-L4}$	16747.2	16746.62
$SLEV_{ΔP4-L4}$	19256.6	19255.83
$ZIKV_{ΔP4-L4}$	16446.0	16445.91
TABV WT	16005.7	16004.91
GBVB WT	14863.0	14862.11
APPV WT	17379.5	17378.38
SPgV WT	17049.3	17048.22

Table S3. Sequences of xrRNA-siRNA constructs and controls. Adapters are bold. Antisense sequences are underlined. siREN, Renilla luciferase-targeting siRNA; siRND, non-targeting control siRNA; S1, strand 1; S2, strand 2; AS, antisense strand; SS, sense strand; 5’ EXT, 5’ extended; X, randomized nucleotide position.

Entry	Sequence (5’ to 3’)
DENV$_{\Delta P4\text{-}L4}$-siREN S1	AGUCAGGCC**AA**GAGCGAAGAGGGCGAGAAAUU
DENV$_{\Delta P4\text{-}L4}$-siREN S1 5’ EXT	AAAGGAAAAUAAAAAAUCAAACAAGGCAAGAAGUCAGGCC**AA**GAGCGAAGAGGGCGAGAAAUU
DENV$_{\Delta P4\text{-}L4}$-siREN S2	UUUCUCGCCCUCUUCGCUCUUGCCAUAGUACGGUAAGAGCUAUGCUGCCUGUGAGCCCs
DENV$_{\Delta P4\text{-}L4}$-siRND S1	AGUCAGGCC**AA**GUGXXUAAXXAACXCAXACUU
DENV$_{\Delta P4\text{-}L4}$-siRND S2	GUXUGXGUUXXUUAXXCACUUGCCAUAGUACGGUAAGAGCUAUGCUGCCUGUGAGCCC
MECDV$_{\Delta P4\text{-}L4}$-siREN S1	AGUCAGACC**AA**GAGCGAAGAGGGCGAGAAAUU
MECDV$_{\Delta P4\text{-}L4}$-siREN S2	UUUCUCGCCCUCUUCGCUCUUGCCACUCAAUGGUUGUAAAGAGUGCUGUCUGUGACAAC
MECDV$_{\Delta P4\text{-}L4}$-siRND S1	AGUCAGACC**AA**GUGXXUAAXXAACXCAXACUU
MECDV$_{\Delta P4\text{-}L4}$-siRND S2	GUXUGXGUUXXUUAXXCACUUGCCACUCAAUGGUUGUAAAGAGUGCUGUCUGUGACAAC
SLEV$_{\Delta P4\text{-}L4}$-siREN S1	AGUCAGGCCAA**AA**GAGCGAAGAGGGCGAGAAAUU
SLEV$_{\Delta P4\text{-}L4}$-siREN S2	UUUCUCGCCCUCUUCGCUCUUUUGCCACCGGAUGUCAGGUAAACGGUGCUGUCUGUAACCUGGC
SLEV$_{\Delta P4\text{-}L4}$-siRND S1	AGUCAGGCCAA**AA**GUGXXUAAXXAACXCAXACUU
SLEV$_{\Delta P4\text{-}L4}$-siRND S2	GUXUGXGUUXXUUAXXCACUUUUGCCACCGGAUGUCAGGUAAACGGUGCUGUCUGUAACCUGGC
ZIKV$_{\Delta P4\text{-}L4}$-siREN S1	AGUCAGGCCG**AA**GAGCGAAGAGGGCGAGAAAUU
ZIKV$_{\Delta P4\text{-}L4}$-siREN S2	UUUCUCGCCCUCUUCGCUCUUCGCCAUGGCACGGAAAAAGCCAUGCUGCCUGUGAGCC
ZIKV$_{\Delta P4\text{-}L4}$-siRND S1	AGUCAGGCCG**AA**GUGXXUAAXXAACXCAXACUU
ZIKV$_{\Delta P4\text{-}L4}$-siRND S2	GUXUGXGUUXXUUAXXCACUUCGCCAUGGCACGGAAAAAGCCAUGCUGCCUGUGAGCC
siREN AS	UUUCUCGCCCUCUUCGCUCUU
siREN SS	GAGCGAAGAGGGCGAGAAAUU
siRND AS	GUXUGXGUUXXUUAXXCACUU
siRND SS	GUGXXUAAXXAACXCAXACUU

Table S4. Masses calculated and found for xrRNA-siRNA constructs and controls. For oligoribonucleotides synthesized with randomized nucleotide positions, the measured masses fall within the expected range, *i.e.*, between the mass of an oligoribonucleotide with G nucleotides at all random positions and the mass of an oligoribonucleotide with C nucleotides at all random positions.

Entry	Mass calculated (g/mol)	Mass found (g/mol)
DENV$_{\Delta P4\text{-}L4}$-siREN S1	10475.5	10474.64
DENV$_{\Delta P4\text{-}L4}$-siREN S1 5' EXT	20642.8	20641.30
DENV$_{\Delta P4\text{-}L4}$-siREN S2	18368.9	18369.20
MECDV$_{\Delta P4\text{-}L4}$-siREN S1	10459.5	10458.57
MECDV$_{\Delta P4\text{-}L4}$-siREN S2	18684.1	18683.92
SLEV$_{\Delta P4\text{-}L4}$-siREN S1	11133.9	11132.9
SLEV$_{\Delta P4\text{-}L4}$-siREN S2	20283.0	20286.57
ZIKV$_{\Delta P4\text{-}L4}$-siREN S1	10820.7	10819.81
ZIKV$_{\Delta P4\text{-}L4}$-siREN S2	18390.0	18390.14
siREN AS	6436.8	6436.09
siREN SS	6901.3	6900.39

Table S5. Sequences of xrRNA-siRNA constructs. Adapters are bold. Antisense sequences are underlined.

Entry	Sequence (5' to 3')
DENV-siREN S1	AGUCAGGCC**AA**GAGCGAAGAGGGCGAGAAAUU
DENV-siREN S2	UUUCUCGCCCUCUUCGCUCUUGCCAUAGUACGGUAAGAGCUAUGCUGCCUGUGAGCCCCGUCUAAGGACGU
MECDV-siREN S1	AGUCAGACC**AA**GAGCGAAGAGGGCGAGAAAUU
MECDV-siREN S2	UUUCUCGCCCUCUUCGCUCUUGCCACUCAAUGGUUGUAAAGAGUGCUGUCUGUGACAACCCAAAUAGUCGAUGGA
ZIKV-siREN S1	AGUCAGGCCG**AA**GAGCGAAGAGGGCGAGAAAUU
ZIKV-siREN S2	UUUCUCGCCCUCUUCGCUCUUCGCCAUGGCACGGAAAAAGCCAUGCUGCCUGUGAGCCCCUCAGAGGACACUGAGU

Table S6. Sequences of mutant MECDV-siREN constructs with stem-loop P4-L4 re-installed. Mutation sites are underlined. MUT, mutant.

Entry	Sequence (5' to 3')
MECDV-siREN S2$_{MUT1}$	UUUCUCGCCCUCUUCGCUCUUGCCACUCAAUGGGGGGAAAGAGUGCUGUCUGUGCCCCCCCAAAUAGUCGAUGGA
MECDV-siREN S2$_{MUT2}$	UUUCUCGCCCUCUUCGCUCUUGCCACUCAAUGCCCCCAAAGAGUGCUGUCUGUGGGGGGCCAAAUAGUCGAUGGA
MECDV-siREN S2$_{MUT3}$	UUUCUCGCCCUCUUCGCUCUUGCCACUCAAUGGCGCGAAAGAGUGCUGUCUGUGCGCGCCCAAAUAGUCGAUGGA

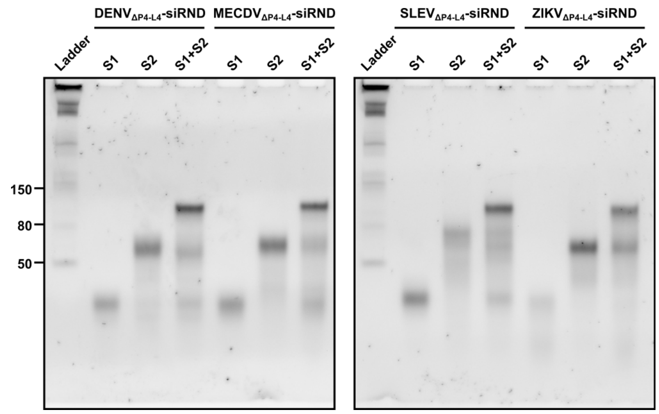

Figure S1. Non-denaturing polyacrylamide gel mobilities of control xrRNA$_{\Delta P4-L4}$-siRNA constructs. Reference bands in the ladder are single-stranded RNA.

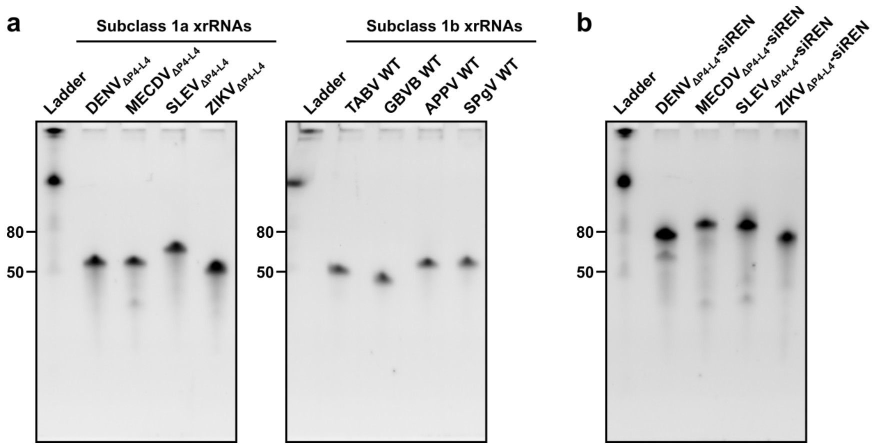

Figure S2. (a) Denaturing polyacrylamide gel mobilities of xrRNA constructs. (b) Denaturing polyacrylamide gel mobilities of xrRNA$_{\Delta P4-L4}$-siRNA constructs. Reference bands in the ladder are single-stranded RNA.

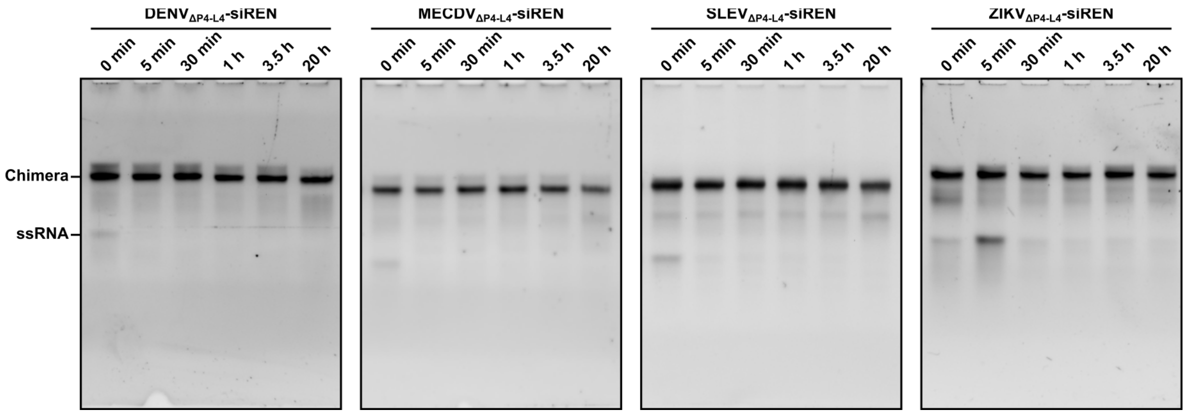

Figure S3. Non-denaturing polyacrylamide gel mobilities of xrRNA$_{ΔP4-L4}$-siRNA constructs with a two-fold molar excess of S1 to S2 following incubation with Xrn1 at 37°C. The excess S1 is labelled as a single-stranded RNA (ssRNA). The length of incubation with Xrn1 is indicated above each lane.

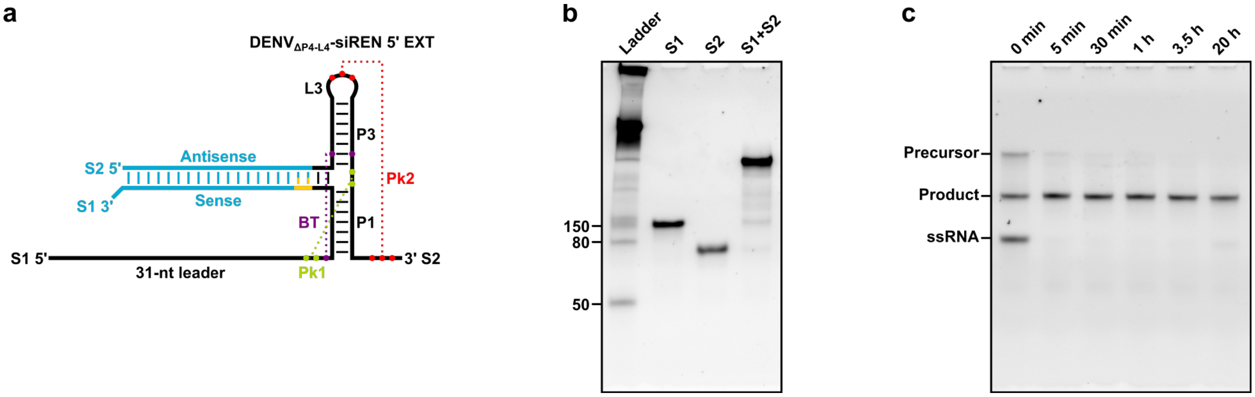

Figure S4. (a) DENV$_{ΔP4-L4}$-siREN 5' extended (5' EXT) construct. A 31-nucleotide (nt), unstructured "leader" sequence at the 5' end of S1 is labelled. (b) Non-denaturing polyacrylamide gel mobility of the DENV$_{ΔP4-L4}$-siREN 5' EXT construct. Reference bands in the ladder are single-stranded RNA. (c) Non-denaturing polyacrylamide gel mobility of the DENV$_{ΔP4-L4}$-siREN 5' EXT construct with a two-fold molar excess of S2 to S1 following incubation with Xrn1 at 37°C. The excess S2 is labelled as a single-stranded RNA (ssRNA). The length of incubation with Xrn1 is indicated above each lane.

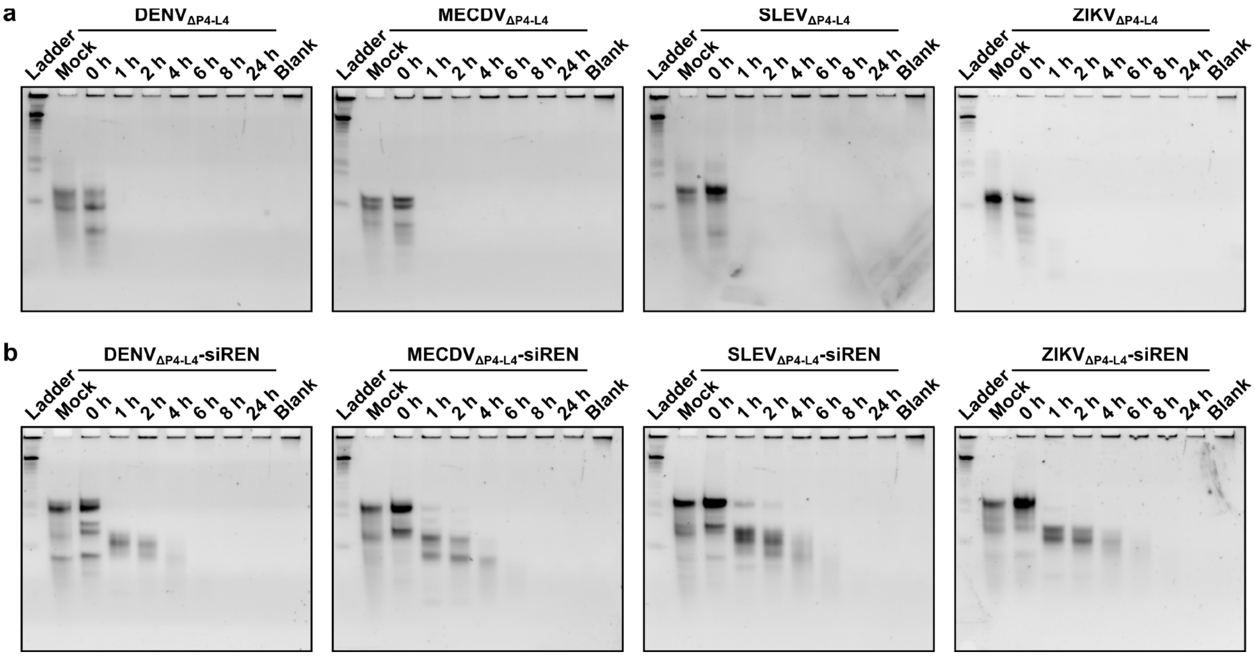

Figure S5. (a) Serum stability assays of xrRNA$_{\Delta P4\text{-}L4}$ constructs in a second batch of mouse serum. (b) Serum stability assays of xrRNA$_{\Delta P4\text{-}L4}$-siRNA constructs in a second batch of mouse serum. The length of incubation in 50% mouse serum is indicated above each lane. Mock is RNA incubated in water at 37°C for 24 h. Blank is 50% mouse serum incubated at 37°C for 24 h. Reference bands in the ladder are single-stranded RNA.

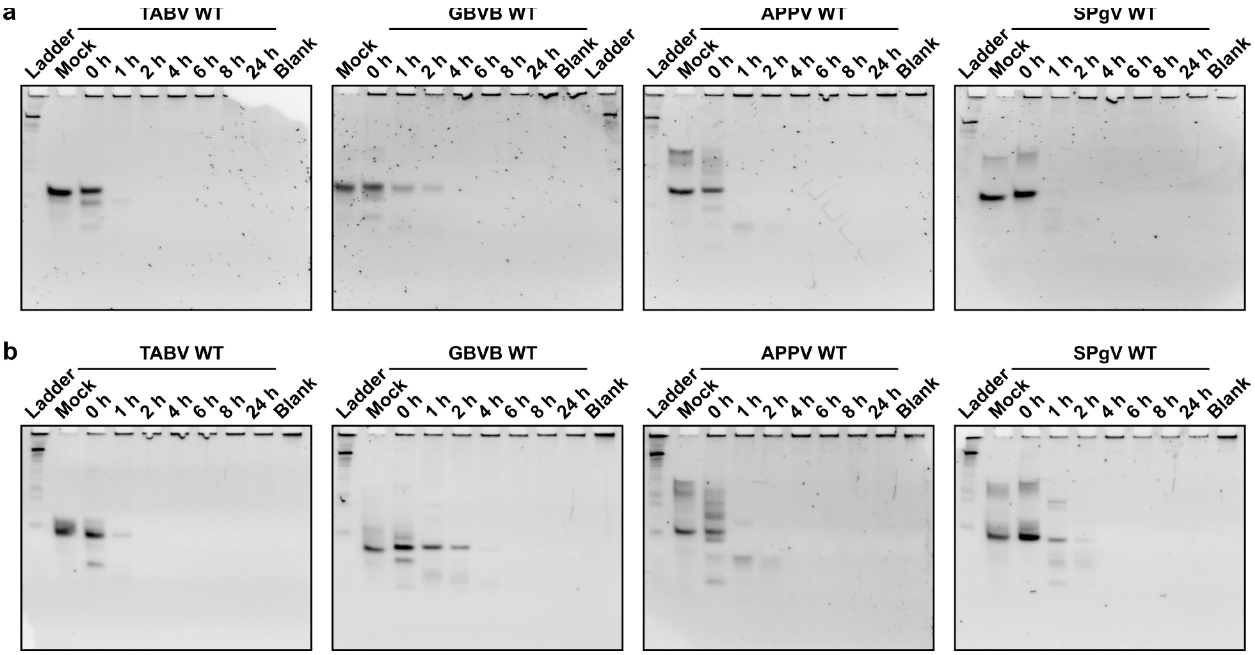

Figure S6. (a) Serum stability assays of xrRNA constructs. (b) Serum stability assays of the same constructs in a second batch of mouse serum. The length of incubation in 50% mouse serum is indicated above each lane. Mock is RNA incubated in water at 37°C for 24 h. Blank is 50% mouse serum incubated at 37°C for 24 h. Reference bands in the ladder are single-stranded RNA.

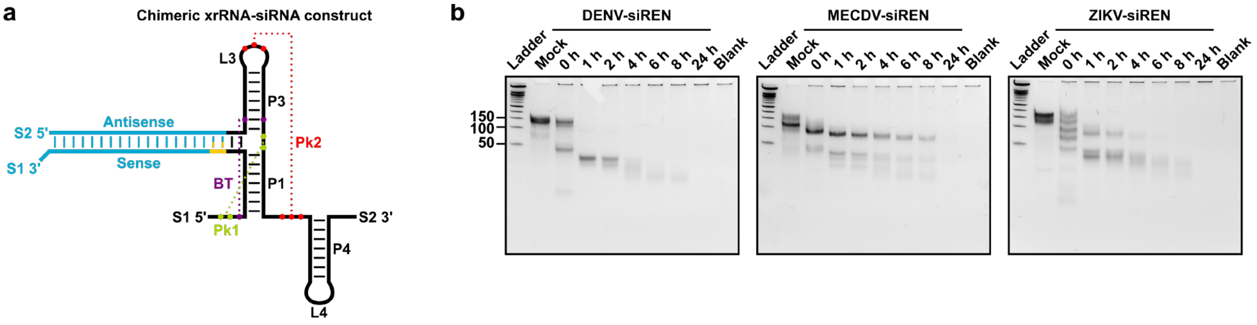

Figure S7. (a) Chimeric xrRNA-siRNA construct. (b) Serum stability assays for xrRNA-siRNA constructs annealed in 1 M NaCl, 10 mM cacodylic acid, pH 7.0. The length of incubation in 50% mouse serum at 37°C is indicated above each lane. Mock is RNA incubated in water at 37°C for 24 h. Blank is 50% mouse serum incubated at 37°C for 24 h. The reference bands in the ladder are double-stranded DNA. Each gel image is representative of three independent replicates.

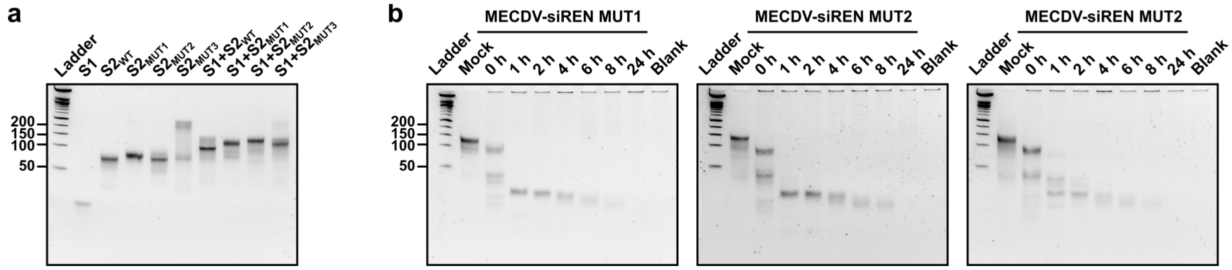

Figure S8. (a) Non-denaturing polyacrylamide gel mobilities of mutant MECDV-siREN constructs. (b) Serum stability assays for mutant MECDV-siREN constructs annealed in 1 M NaCl, 10 mM cacodylic acid, pH 7.0. The length of incubation in 50% mouse serum at 37°C is indicated above each lane. Mock is RNA incubated in water at 37°C for 24 h. Blank is 50% mouse serum incubated at 37°C for 24 h. In (a) and (b), the reference bands in the ladder are double-stranded DNA.
